# Overview of DNA Self-Assembling: Progresses in Biomedical Applications

**DOI:** 10.3390/pharmaceutics10040268

**Published:** 2018-12-11

**Authors:** Andreia F. Jorge, Ramon Eritja

**Affiliations:** 1 Coimbra Chemistry Centre (CQC), Department of Chemistry, University of Coimbra, Rua Larga, 3004-535 Coimbra, Portugal; andreiaj@qui.uc.pt; 2 Institute for Advanced Chemistry of Catalonia (IQAC-CSIC), Networking Center on Bioengineering, Biomaterials and Nanomedicine (CIBER-BBN), Jordi Girona 18-26, E-08034 Barcelona, Spain

**Keywords:** DNA self-assembling, gene delivery, drug delivery, protein delivery, theranostics, nanomedicine

## Abstract

Molecular self-assembling is ubiquitous in nature providing structural and functional machinery for the cells. In recent decades, material science has been inspired by the nature’s assembly principles to create artificially higher-order structures customized with therapeutic and targeting molecules, organic and inorganic fluorescent probes that have opened new perspectives for biomedical applications. Among these novel man-made materials, DNA nanostructures hold great promise for the modular assembly of biocompatible molecules at the nanoscale of multiple shapes and sizes, designed via molecular programming languages. Herein, we summarize the recent advances made in the designing of DNA nanostructures with special emphasis on their application in biomedical research as imaging and diagnostic platforms, drug, gene, and protein vehicles, as well as theranostic agents that are meant to operate in-cell and in-vivo.

## 1. Introduction

Nowadays, there is an increasing demand for developing predictive, preventive, and non-invasive patient-centered medicines, ideally combining diagnosis and therapeutics in one single device to enabling early diagnosis, precise treatment, and management of a specific disease, with power to leverage the quality of medical care [1]. Thus, the concept of theranostics has emerged to enclose this modern approach that is expected to address medical needs especially in the treatment of life-threatening diseases [2,3].

In recent decades, researchers developed artificial molecular assemblies at the nanoscale from biomolecules such as lipids, peptides, proteins, DNA, or synthetic organic molecules, such as linear, branched, dendritic polymers, lipid analogs or synthetic inorganic molecules, such as gold nanoparticles or quantum dots with the goal of emulating biomolecular engineering and providing suitable tools to operate within living cells [4,5,6,7,8,9,10,11]. These molecular assemblies have broad impacts in diverse disciplines, especially in synthetic biology, molecular analysis, biocomputing, drug delivery, cellular imaging, and electronics, among others. Many of these nanotechnology systems are currently under clinical trials and some are already approved by US Food and Drug Administration (FDA) for human applications [12]. The success of the nanotechnology-based systems designed for drug delivery requires some key ingredients including the ability to: (i) overcome multidrug resistance; (ii) offer protection to degradable therapeutics; (iii) mitigate cytotoxicity and drug side effects; (iv) increase solubility of hydrophobic drugs; (v) control drug release; (vi) bypass cell barriers; and (vii) enter in target cells. These requirements are also extended to the fields of molecular analysis and cell imaging since analytic nanodevices should internalize cells to bind specifically to biomarkers or antibodies and carry with them, e.g., hydrophobic fluorescent dyes or quantum dots for transducing biological signals. In cell imaging, high efficient nanoparticles are thus a requisite to obtain high contrast images, otherwise greater concentration of probe is required which limits the application of nanoparticles due to their cytotoxicity [13,14]. Despite all the compelling advances taken in this field, the nanotechnology-based systems engineered so far are still not fully successful under in vivo settings and further improvements are urgently necessary. For instance, cationic polymers and dendrimers are inherently cytotoxic and their instability under physiological conditions promotes adverse side effects and affects drug delivery efficiency [15,16]. Surface functionalization of nanoparticles represents an opportunity to reduce unspecific interactions between nanoparticles, but preserving colloidal stability under physiological conditions is particularly challenging [17,18].

DNA-based nanotechnology has emerged as a new route to fabricate biocompatible well-defined scaffolds, given their biological origin, with unparalleled structural precision and tailorability that allows the construction of a large range of self-assembled structures in a bottom-up manner [19,20,21]. By virtue of its high programmability, the opportunity to organize small molecules, DNA or RNA, or even nanomaterials at a precise stoichiometry and distribution over a static or dynamic scaffold that can range from one-dimensional (1D) to three-dimensional (3D) arrangements, offers high ability to increase molecular recognition in living cells, build versatile templates to understand biomolecular interactions, and sharpen cell imaging. All these features open up new opportunities to advance the growing of nanotheranostics [22,23]. To demonstrate the success and potential of DNA nanotechnology in the development of DNA-based imaging and sensing probes and in the prototype of smart delivery systems, we review the recent progress made in this direction. 

Firstly, the origin and development of DNA nanoscaffolds are briefly introduced with special emphasis on most relevant static and dynamic architectures engineered. Secondly, we compile the recent research performed to create DNA-based strategies for enabling high-resolution imaging of biomolecules inside living cells or cell-like environments so as cell lysates and fixed cells. Next, the potential of DNA biosensors as analytical tools capable of quantitatively detect the presence of disease-associated biomarkers are gathered and compared. New perspectives for the delivery of drug, therapeutic oligonucleotides, and therapeutic proteins are introduced while stressing the potentialities of the different DNA-based strategies. Finally, special attention is dedicated to highlighting the interesting therapeutic outcomes that arise from the combination of programmable functional DNA nanoscaffolds with photodynamic and/or photothermal agents. 

## 2. Initial Development of Static DNA Nanostructures 

**Figure 1:**
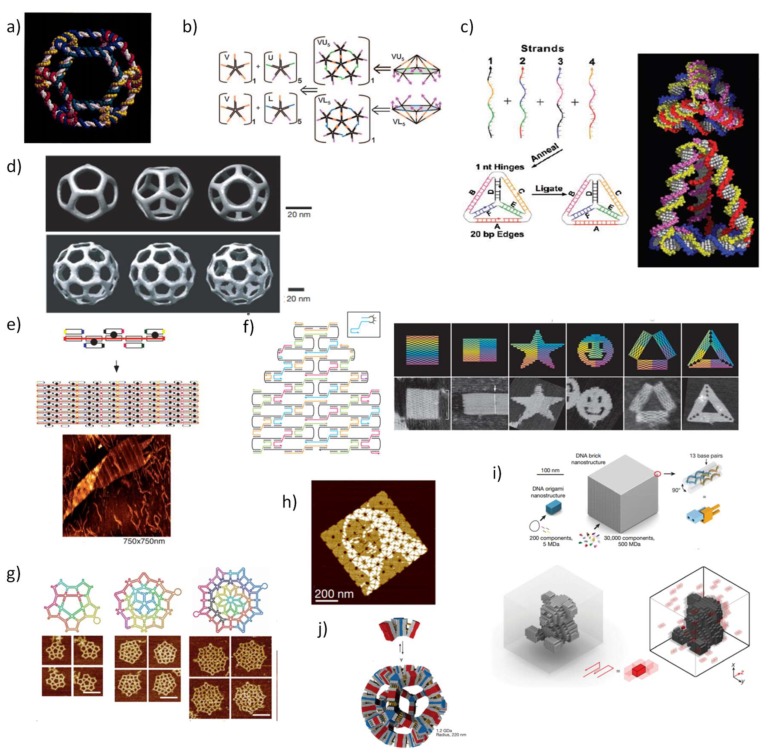
Static DNA nanostructures. (**a**) DNA-truncated octahedron assembled by DNA tiles, adapted with permission from [30], (**b**) DNA icosahedron, adapted with permission from [31], (**c**) DNA tetrahedron adapted with permission from [32,33] and (**d**) multiarm DNA tiles for assemble 3D polyhedral structures, adapted with permission from [34]. (**e**) Two-dimensional (2D) aperiodic patterned DNA lattice self-assembled by a large number of shorter synthetic oligonucleotides around a longer single-strand scaffold, adapted with permission from [35]. Copyright (2003) National Academy of Sciences, U.S.A. (**f**) Rothemund’s 2D origami, adapted with permission from [37]. (**g**) Examples of complex wireframe DNA origami nanostructures assembled by multi-arm junction vertices, adapted with permission from [42]. (**h**) Two-dimensional (2D), adapted with permission from [53] and (**i**) and (**j**) three-dimensional (3D) nanostructures self-assembled from DNA bricks, adapted with permission from [54,55].

In addition to the recognized fundamental role of DNA in biology as the repository of genetic information, in the past two decades, DNA has demonstrated to be an excellent backbone for the construction of biocompatible nanostructures. The precise and predictable nature of the Watson and Crick base-pairing rules is the most salient feature of the success of DNA nanotechnology, coupled with its well-established structural parameters, in which under physiological conditions, the double-stranded (ds) helix mainly adopts the relatively stiff B-form conformation, with about 2 nm in diameter and 3.4 nm per helical turn [24,25]. The remarkable control over the intermolecular interactions between DNA strands confers a valuable tool for programming by sequence the correct duplex formation to create unique scaffolds. N. Seeman, in a seminal work [26], set the bases for the DNA tile assembly technique motivated by the idea of creating artificial crystals. In this design, four single DNA strands were joined together at a single point to self-assemble into “branched junctions” to form a four-arm junction, inspired by the Holliday junction found in nature [27]. Soon after, the same authors reported the design and characterization of 2D-crystaline forms of DNA, whereby the self-assemble of synthetic double-crossover (DX) molecules is programmed by sticky-ended associations following base-pair complementary principles [28]. In a remarkable work, multi-armed junctions were used to assemble the first discrete 3D structure, a DNA cube [29]. Meanwhile, other interesting discrete 3D-structures were created including a truncated DNA octahedron (Figure 1a) [30], a modular icosahedron (Figure 1b) [31] and a DNA tetrahedron (Figure 1c) [32,33]. In this latter work, Turberfield and colleagues developed a rapid methodology for producing DNA tetrahedral nanocages simply by mixing four DNA strands at equimolar concentration covalently bound at the vertices, generating small nanocages with different edge length and structure rigidity. Mao and coworkers extended the formation of tile-based nanostructures by assembling diverse 3D polyhedral structures with identical arms and tunable flexibility based on multiple three-point-star DNA tiles (Figure 1d) [34].

In 2003, Reif and coworkers were the first to describe the construction of an aperiodic patterned DNA lattice based on the self-assembly of multiple shorter synthetic oligonucleotides around a longer single-strand (ss-DNA) scaffold (Figure 1e) [35]. Next, Shih et al. published a method to assemble a DNA octahedron, where a single-stranded DNA molecule with 1669-nucleotide (nt)-long amplified by polymerases folds into an octahedron structure by the action of five 40-mer complementary strands after thermal annealing [36]. These two works provided the fundamental concepts and a strong basis for the development of what is acknowledged today as DNA origami structures.

In 2006, a well-defined, versatile, and easy to implement technique to assemble DNA origami was reported by P. Rothemund [37], whereby a long, single-stranded scaffold, typically the commercially available M13 bacteriophage genome DNA with approximately 7000 nt), is folded via thermal annealing by hundreds of short ‘staples’ strands (~200 nt) in a single step to create intricate DNA platforms. In this work, Rothemund designed high-yield flat 2D DNA objects with roughly 100 nm of diameter, where the smiley face was the first to be assembled (Figure 1f). Two strategies of design are distinguished in DNA origami methodology, the lattice-based origami and the wireframe-origami. This distinction is used to discern the arrangement of the helices within DNA origami. In lattice-based origami, DNA helices are closely packed in a lattice-like packing following square, honeycomb, hexagonal, or hybrid lattices [38,39,40], whereas in wireframe-origami, the packing between DNA helices is avoided and consequently porous structures are created. 

In the case of wireframe-origami, Yan group used DNA four-arm junctions as vertices of a DNA network to generate DNA gridiron structures [41], and later, more intricate structures with multi-arm junctions were built (Figure 1g) [42]. Likewise, DNA lattice-based origami originated also 3D objects. For instance, a hollow 3D-megadalton-sized DNA box with a controllable lid was assembled from six flat 2D-DNA faces stitched together by linking staple strands [43]. Using a different approach, termed multi-layered origami, 3D shapes were formed as pleated layers of helices constrained to a honeycomb [40]. Furthermore, a hollow 3D DNA tetrahedron was constructed by using one single scaffold strand that runs through all adjacent triangles connected by single-stranded staples to define the edges [44]. 

This bottom-up fabrication complemented by the development of computational tools for modeling and visualization of DNA nanostructures promptly facilitated complex DNA structures available to a wider scientific audience. Today, an ever-growing number of researchers are exploiting the programmable properties of DNA nanotechnology expanding the use of rational nanomachines at nanometer scale with enormously high precision in a vast number of applications [45,46,47]. One of the first software to design DNA nanostructures was the GIDEON software [48], developed by Seeman and colleagues and published in 2006, but had little impact. SARSE [49] was then the first available user-friendly software to design DNA origami released after Rothemund´s methodology, with a 3D generator that facilitates the construction of the 3D atomic model for a desirable structure that can be visualized with a molecular visualization system, and an oligotracker to edit and save the list of staple-strands and their sequences. Soon after this proposal, other structure predicting software was developed which is widely recognized today and used to ensure the correctness of the design, such as caDNAnano [50] and CanDo [51]. 

In 2012, Yin and coworkers designed a novel type of DNA tile using a short synthetic single-stranded DNA to form a 3D structure, named as DNA bricks [52]. In particular, each 32-nt brick represents a modular component, able to interact with four local neighbors and can be replaced or added in an independent manner. This modularity confers higher versatility than origami technique since the lack of a long ssDNA enables engineering a number of outstanding DNA objects with high atomic masses and larger sizes in 2D (Figure 1h) [53] or 3D shapes (Figure 1i,j) [54,55]. 

## 3. Dynamic and Functional DNA Nanostructures 

The obvious biocompatibility and high programmability of DNA objects sparked interest in their use in biological applications. Applying the fundamental concepts exploited in the formation of such large spectrum of static DNA nanostructures, researchers exploited the modularity of the DNA nanostructures to encode sensing, reporting and targeting components. The design of dynamic DNA nanomachines having these functions almost always requires the integration of mechanical and chemical properties to provide local motion, flexibility, conformational changes, chemical reactivity, and biochemical responsiveness to specific triggers. The advanced chemistries used for the functionalization of oligonucleotides provided great knowledge and opportunities to combine synthetic organic fluorophores, gold nanoparticles, aptamers, antibodies, proteins, lipids, and therapeutic modules with single-strand DNA oligonucleotides that are captured by complementary strands located in the interface of DNA devices [56,57,58,59,60,61,62,63]. Aptamers are non-toxic single-stranded DNA or RNA molecule with high affinity and specificity to their target molecules with a structure rigorously selected and optimized by a procedure known as systematic evolution of ligands by exponential enrichment (SELEX) [64]. Some antibodies and proteins, like aptamers, are ideal candidates to act as recognition modules able to direct the interaction of DNA nanodevices with specific cells or tissues.

**Figure 2:**
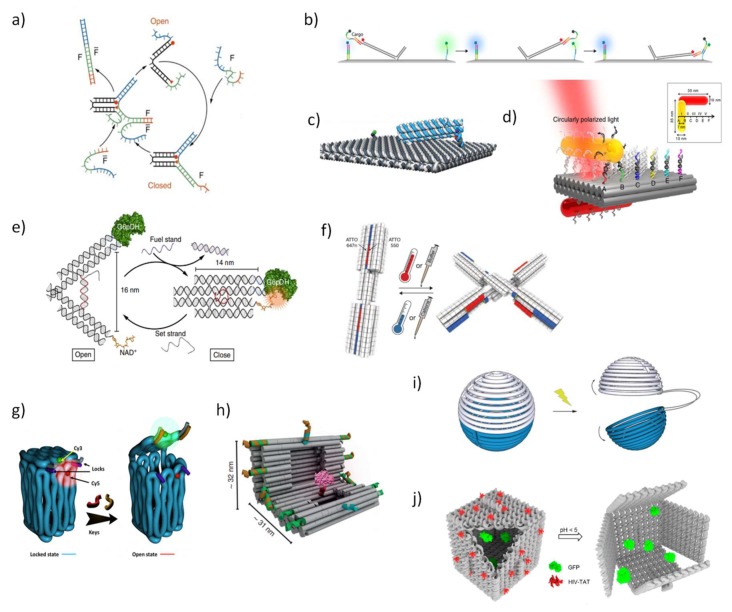
Examples of dynamic DNA-based nanostructures. Reconfigurable strand systems change their configuration upon DNA strand displacement such as (**a**) Yurke’s molecular tweezers, adapted with permission from [66]. More sophisticated DNA motors can transport DNA strands (**b**) and (**c)**, adapted with permission from references [71,72] and (**d**) gold nanorods, adapted with permission from [73] in response to specific cues transporting molecular cargo along a supramolecular platform. Target recognition modules that induce shape-changing events and signal transducers in one single device leading to measurable readouts are useful as sensors. For example, the mechanical control can be dictated by (**e**) G6pDH/NAD+ enzyme-cofactor pair, adapted with permission from [75] or via (**f**) DNA base pairing controlled by temperature and salt conditions, adapted with permission from [77]. DNA nanodevices able to encapsulate cargo with reconfigurable shapes addressed by components sensitive to complementary DNA strands, in (**g**) and (**h**) [78], (**i**) light [82] and (**j**) pH [84], adapted with permission from the given references.

Among DNA-based dynamic devices, reconfigurable strand systems are powerful responsive devices that change configuration, as for example through DNA strand displacement [65], in response to diverse biomolecules and environmental conditions. For example, Yurke and coworkers in a pioneer work devised a functional DNA tweezer, in which DNA supplies not only the main scaffold but also the “fuel” for motion, produced by hybridization and strand displacement reactions (Figure 2a) [66]. Owing to its simplicity, other molecular motors approaches have been engineered to generate multiple strand displacement to create complex chain cascades and provide sensing and amplified signals [67,68,69]. In the field of in-cell diagnosis and imaging, special attention has been devoted to the great potential of signal amplification offered by in situ hybridization chain reaction techniques to detect low abundant biomolecules [70]. More intricate assemblies, outside of cellular environment, have been also explored to transport DNA cargo strands on a supramolecular platform via an extended tether arm, providing an efficient, guided and fast transfer of DNA cargo over long distances controlled by electric field (Figure 2b) [71,72]. Other than DNA cargo, origami DNA motors have the ability to transport, for example, gold rods along a track defined by successive binding sites (Figure 2c) [73] or transport a photo-responsive co-factor attached to a swinging arm, whose location is controlled by light that selectively activates or inhibits the enzyme cascade reaction disposed in the origami surface [74].

Ideally, a biomolecular biosensor based in dynamic DNA nanostructures should be composed by a target recognition module that induces shape-changing events and signal transducers in one single device. A tweezer-like DNA nanoreactor with the ability to modulate the activity of a G6pDH/NAD^+^ enzyme-cofactor pair via mechanical control was designed (Figure 2e) [75]. In response to external stimuli, as regulatory DNA strands, several cycles of enzyme inhibition and activation were measured in real-time by fluorescence (Föster) resonance energy transfer (FRET). In a DNA-based light-driven plasmonic system, light induces dynamic conformational changes by interfering in the hybridization of azobenzene-modified DNA oligonucleotides, and the interchange between open and locked states can be optically tracked and efficiently retrieved [76]. In a remarkable work, Dietz and co-workers [77] created reconfigurable shape-complementary DNA objects whose weak base-pairing is regulated by changes in ion conditions or temperature, with two organic dyes to enable a FRET-based readout (Figure 2f). Diverse robust DNA-based stimuli-response objects have been drawn with high structural complexity at a nanometer scale and with fast and accurate responses, however, major challenges in achieving continuous control over dynamic conformations still remain and importantly the harder task is to control their function in physiological conditions.

In addition, the delivery of nanostructures into cell culture and animals will require devices that are stable and able to protect the cargo. The design of reconfigurable and addressable objects that can encapsulate molecules and then release them in response to environmental cues has been pursued for a long time in the DNA-nanotechnology field. With this goal in mind, a 3D DNA box origami, smaller than the one reported by Jahn and coworkers [43], was programmed to be repeatedly closed and opened though consecutive DNA strand displacement events (Figure 2g) [78]. Other studies have explored not only other shapes of DNA containers but also different types of environmental cues, such as DNA nanocages sensitive to double-helix locks (Figure 2h) [79], or DNA icosahedron containers sensitive to bacterial cell signaling [80], or a DNA nanorobot containers sensitive to cell surface receptors [81], or DNA origami spheres or nanocages sensitive to light (Figure 2i) [82,83], or DNA nanocages sensitive to pH changes (Figure 2j) [84]. Confining proteins in a DNA-based engineered environment has been used to elucidate functional and structural properties of protein–protein interaction through single-molecule methods while providing protection against proteases. To this end, several DNA containers were developed to fix and encapsulate proteins [79,85,86].

## 4. Stability of DNA Nanostructures in Biological Environments

The lack of structural integrity of nucleic acid devices may compromise their applicability in biomedical studies. To test the structural integrity of nucleic acid devices in biological conditions generally cell lysates and standard cell culture media supplemented with serum containing nucleases, specifically DNases, are used for simulating the environment found in biological fluids and inside cells. For example, Meldrum and coworkers [87] assembled DNA origami with distinct shapes, sizes, and probes and tested their stability in cell lysates of normal and cancerous cell lines. Results confirmed the stability of DNA origami nanostructures that could be extracted from lysates and characterized after 12 h of incubation, in contrast to long ss- and ds-DNAs that were readily degraded. Atomic force microscope and transmission electron microscope images further guaranteed that DNA nanostructures were fully intact after incubation and separation from cell lysates.

DNA nanostructures are commonly assembled in TAE buffer supplemented with moderate concentration of divalent ions (Mg^2+^) for screening the electrostatic repulsion existent between neighboring DNA tiles. Altering buffer conditions may affect DNA assembling process leading to shape distortion and structural collapse [88,89]. Perrault and coworkers [89] performed a systematic characterization of the stability of three different DNA nanostructures to mammalian tissue culture conditions. They found that structural integrity strongly correlates with origami design, the presence of divalent ions Mg^2+^ and the level of nuclease activity present in FBS used as a medium supplement. In particular, low volumes of FBS ranging from 1 to 2.5% (*v*/*v*) had little ability to digest a 5 nM concentration of DNA nanostructure over 24 h. Among the three structures tested, the DNA nano-octahedron, the six-bundle nanotube and the 24-helix nanorod, only DNA nanotube appeared to be stable in physiological cation concentration after 24 h at 37 °C. To determine the effect of DNases in nanostructure stability, Castro et al. [51] in a complete study assessed the degree of nanostructure degradation of three test multilayer structures with honeycomb lattice packing and provided information on the conditions under such objects are expected to remain stable. Firstly, they observed that all structures can be safely incubated at 37 °C for 24 h, and in addition, no structural alterations were observed when incubated at room temperature in different buffering media. Finally, exposing the test objects to various nucleases showed that only DNase I and T7 endonuclease I were able to degrade the tested structures. 

All of this evidence has demonstrated that DNA nanostructures resist degradation better than the constitutive ss- or ds-DNA, nevertheless their short-term stabilization in low-salt and nuclease-rich physiological media may hamper biological applications. Therefore, research efforts have been conducted to retain their structural integrity over longer time scales in physiological conditions. With this in mind, Sleiman and colleagues [90] modified the ends of a DNA prismatic cage with hexaethylene glycol and hexanediol. These simple modifications were shown to increase the stability of DNA strands towards nuclease and increasing the lifetimes to 62 h in serum. Furthermore, Shih and coworkers [91] coated DNA nanostructures with positively charged oligolysine-polyethylene glycol (PEG) copolymer. Coated nanostructures are significantly stabilized against nuclease degradation at physiological divalent-ion concentrations and no distortion of the 3D arrangement was detected, thus overcoming two major challenges in their in vivo application. In addition, preliminary mice experiments indicated a modest enhancement in circulation and biodistribution of coated nanostructures in comparison to the uncoated ones. The coating of DNA nanostructures was also performed with other protonable cationic agents, such as ethylenediamine, to achieve pH-responsive DNA assembly, overcome electrostatic repulsion, and circumvent nuclease degradation [92]. The well-known polycations chitosan and linear polyethylenimine were also exploited as coating agents for three different DNA nanostructures, formed spontaneously through electrostatic interactions [93]. This study revealed a significant contribution for the stability of wireframe DNA origami nanostructures ensuring their stability in culture media up to a week. The same strategy was used to increase the stability of DNA nanoprism with the natural spermidine. Importantly, these modifications have shown to increase not only the thermal stability and the enzymatic resistance when compared to Mg^2+^-assembled DNA nanoprism but also enhance cellular uptake efficiency in tumor cells [94]. 

## 5. DNA Nanostructures for Biomedical Research

Substantial efforts have been carried out to leverage the use of DNA nanodevices to combine diagnostic and therapeutic abilities in a single powerful platform, enabling diagnosis, drug delivery, and treatment response monitoring, and hitherto important progress has been drawn toward this desirable combination. In this section, the recent advances made in biomedical applications will be summarized, highlighting the key achievements in the development of DNA-based imaging probes, prototypes of dynamic biosensors, and smart therapeutic systems. This review covers mainly the strategic design concepts exploited to accomplish the hard task of operating inside live cells and perform high-throughput analysis in crowded physiological fluids.

### 5.1. DNA Nanostructures for In Vitro and In Vivo Bioimaging

DNA nanotechnology has long been motivated by the purpose of constructing tools for cellular imaging that might contribute for the understanding of functional and morphological details of biological systems. The suitability of DNA nanostructures for building these tools arises particularly from their capacity to precisely incorporate different functional species with stoichiometry control at nanometer scale and from the straightforward manner in which they can be programmed to hybridize exogenous and endogenous nucleic acids, not to mention their inherent biocompatibility. This consequently sparked the use of various DNA origami architectures as imaging platforms to calibrate super-resolution microscopes as well as templated matrices to tune the optical properties of plasmonic systems, improving the engineering of powerful near-field and far-field optical techniques highly valuable in molecular diagnosis and bioimaging.

**Figure 3:**
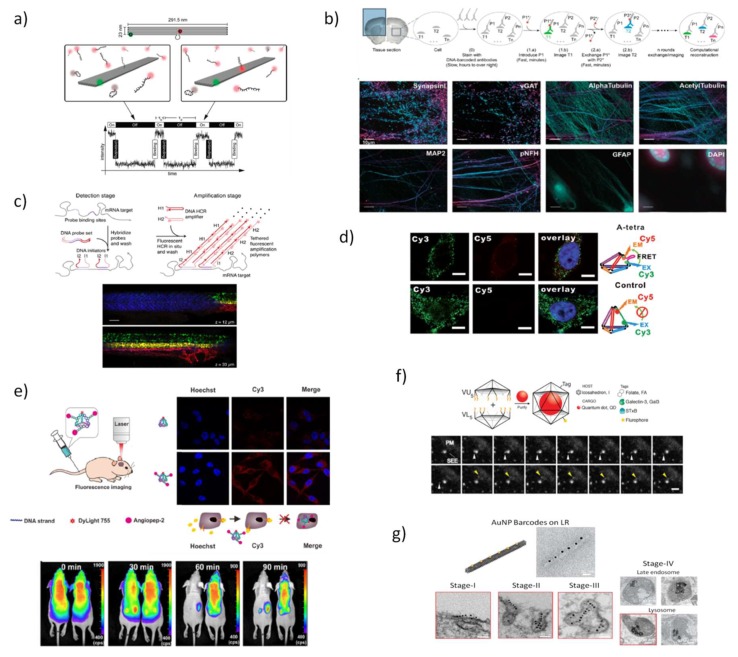
Examples of DNA nanostructures for in vitro and in vivo bioimaging. Fundaments of super-resolution microscopy technique (DNA-PAINT) (**a**) that exploits the complementarity of DNA sequences to provide transient binding of fluorescently labeled DNA strands to DNA docking strands protruding DNA-based nanostructures, adapted with permission from [96] and (**b**) multiplexing DNA Exchange Imaging (DEI) in complex biological samples, adapted with permission from [99]. Initially, different targets (T1,…Tn) are labeled with antibodies conjugated to orthogonal DNA strands (P1,…Pn) to forming imager strands (P1*, …Pn*). These imager strands can be rapidly and sequentially washed away by buffer exchange allowing efficient multiplexed in situ imaging. Hybridization chain reaction (HCR) in situ (**c**) provides also multiplexed imaging by exploiting the complementarity of RNA probes to mRNA targets triggering chain reactions to produce fluorescent amplified RNA-based polymers, adapted with permission from [70]. Reconfigurable DNA TDN (**d**) is able to detect intracellular ATP in living cells, adapted with permission from [109]. A fluorescently labeled DNA TDN (**e**) proved to be a powerful biocompatible imaging probe for brain tumor-targeting, following the cellular uptake in an in vitro BBB model and exhibits biodistribution in mice, adapted with permission from [113]. DNA octahedron (**f**) encapsulating quantum dots [119] and (**g**) DNA nanorods barcoded with AuNPs [120] were used as probes to imaging endocytic pathways by following the conjugated photomaterial during the cellular uptake in fixed cells. Adapted with permission from [119] and [120].

In a seminal work, Simmel and coworkers [95], used a rectangular DNA origami with two embedded fluorescent dyes at specific positions with a defined separation, by chemically modifying some origami staple strands at the 5’-end, to be imaged using three types of super-resolution far-field fluorescence microscopes. Shortly after this proposal, an exciting new type of super resolution fluorescence technique was developed by the same group [96], the so-called DNA point accumulation for imaging in nanoscale topography (DNA-PAINT). This new approach exploits the fundamental reversibility of DNA hybridization that is tunable by changing the nucleotide sequence or length and the concentration of the complementary fluorescent strands. In detail, there is a stochastic transient binding of fluorescently-tagged oligonucleotides, the ‘imager’ (9–10 nucleotides long) to complementary ‘docking’ strands—that protrude from long rectangular DNA origami—leading to a transient switching of the fluorescence signals between the ON- and OFF-states, thus allowing individual target sites to be imaged with sub-10 nm spatial resolution (Figure 3a). The high specificity of DNA hybridization enables multiplexing capabilities and wide adjustability of ON- and OFF-times. DNA-PAINT was further leveraged to generate multicolored images delineating different components of fixed human cells, by using short and orthogonal oligo-fluorophore that bind to the complementary docking sequences in a DNA origami or to antibodies located in cellular organelles, which can be removed by a simple washing step after imaging (Exchange-PAINT) [87,97]. The overlay of all cycles of imaging obtained from the same biological sample produces a multicolored image. This technique was tailored to the imaging of fixed cells by targeting proteins with antibodies attached to the docking strands, achieving four-colored super-resolution images of cellular microtubules, mitochondria, the Golgi complex, and peroxisomes [97]. Agasti et al. [98] validated the performance and orthogonality of 52 DNA sequences directly conjugated to antibodies and they successfully achieved a nine-target super resolution imaging in fixed biological samples. Recently, the application of Exchange-PAINT technique was extended to other super resolution microscopy systems and confocal microscopes. The authors proposed the use of semi-transient and dense target labeling with fluorophore-tagged complementary imager strands rather than transient, thus allowing easy strand exchange, fast image acquisition, and deeper sample diffusion. Multiplexed eight-target imaging in fixed neurons (Figure 3b) and frozen mouse retina tissue sections was collected using confocal microscopy [99]. To minimize the background caused by non-binding DNA “imager” strands, DNA-PAINT was combined with FRET principles, using two imager strands functionalized with a donor and an acceptor dye to generate FRET signal [100,101]. As a result, FRET-PAINT enables the measurement of distances in the range of 1–10 nm, thus improving the spatial-resolution obtained with DNA-PAINT. DNA-based super-resolution imaging provides also quantitative target detection that allows counting dye-labeled DNA probes with high accuracy and precision [102], and even allows direct single-molecule detection and quantification of synthetic and endogenous miRNAs as well as discrimination between single molecule polymorphisms [103]. In this regard, the interest of mapping the subcellular distribution of endogenous mRNAs due to their implication in diverse diseases has stimulated the development of new fluorescence-based techniques that efficiently report the localization of such low abundant and short RNA sequences. 

One of the earliest forms of functional nucleic acid nanodevices enabling real-time tracking of short RNA in living cells were simple fluorescently labeled antisense probes, known as DNA molecular beacons [104,105]. However, these small DNA devices are not efficient in cell internalization, exhibit low stability in physiological conditions, may interfere in cellular function and their capacity to detect simultaneously multiple intracellular species is still challenging. Using a different approach, multiplexed RNA imaging has been carried out by molecular probes based on a triggered hybridization chain reaction (HCR) [68,70,106] that takes place within cells and originate a nicked ds-DNA polymer, yielding an amplified signal approximately 200-fold brighter labeling of endogenous DNA or RNA than using single-oligonucleotide fluorescent labeling, as for instance fluorescence in situ hybridization (FISH), and permitting the simultaneous mapping of up to five target mRNAs through fluorescence microscopy (Figure 3c). Wu et al. [107] were the first to detect mRNAs in living cells by using a nonenzymatic hairpin DNA cascade amplifier (HDCA), where the endogenous mRNA triggers the assembly of two hairpins duplex resulting in a fluorescent signal and also catalyzes the regeneration of multiple hairpin duplexes in repeated cycles leading to the amplification of the signal produced from one mRNA target. 

Other related approaches were devised recently to enable the imaging of miRNAs inside live cells. Zhou et al. [108] prepared a dual-color encoded DNA tetrahedron (TDN) modified with two hairpin sequence probes on the two opposite edges that specifically recognize miRNA-21 and miRNA-155 in human breast cancer cells. The binding of the target miRNAs to the corresponding complementary regions of the hairpins alter their initial positions, increasing the separation between fluorophores from the quenchers, which results in fluorescent signals with different emission wavelengths for multiplexed detection of the targets. This reconfigurable DNA TDN showed to be stable in physiological conditions and able to internalize cells. Further dynamic fluorescent DNA nanostructures have been also exploited to the real-time sensing of specific environmental cues inside living organisms. Pei et al. [109] created a series of reconfigurable DNA TDNs composed by dynamic sequences into one or two edges that are responsive to specific molecular signals including protons, ATP, and mercury ions (Figure 3d). Since FRET is dependent on the distance between the donor (fluorophore) and acceptor (quencher), the target-induced conformation changes in the dynamic nanostructures are detected in living cells by variation in fluorescence intensity. For a similar purpose, a simple yet powerful pH-triggered DNA nanomachine called the I-switch was proposed by Kirshnan group [110,111], and relied on FRET to map autonomously the spatiotemporal pH changes during the maturation of endosome in nematode *Caenorhabditis elegans*. This device was based on Yurke´s DNA tweezers, where two double helices are attached by a flexible hinge and a pH-sensitive i-motif quadruplex structure able to open and close the tweezers. Once inside endosome and trafficked from early endosome to lysosome, I-switch senses a difference in pH ranging from 6 to 5, resulting in quantifiable fluorescence readout, and thereby, yield an indirect measurement of the pH. Using this pH-sensitive nanomachine coupled to proteins such as tranferrin or furin, it is possible to track multiple endocytosis pathways inside the same cell [112]. 

The use of fluorescent modules attached to DNA nanostructures is also helpful to temporally determine the release of the cargo and map the distribution of the DNA nanostructures in the living organisms. Tian et al. [113] designed a DNA tetrahedron (TDN) for brain-targeting imaging, modified with the fluorescent dye DyLight 755 and a 19-mer peptide derived from human Kunitz domain of aprotinin, the angipep-2 (ANG) (Figure 3e). This modification conferred high binding efficiency with low-density lipoprotein receptor-related protein-1 present in blood-brain barrier (BBB) and glioma. In vitro and in vivo studies confirmed the ready ability of ANG-TDNs probe to cross the BBB model and BBB of normal mice, respectively, and also to provide stronger fluorescent signal inside U87MG human glioblastoma xenograft in mice. Kirshnan and coworkers [114] used a DNA wireframe icosahedra to release small fluorescent polymers confined in a reservoir located in the internal void of the nanostructure that can be activated by light stimulus for in vivo administration giving unprecedented spatiotemporal control over the delivery of the cargo. This technology can estimate the concentration of small molecules released after photoactivation as well as determine the exact location at which uncaging of molecules takes place. Imaging of *Caenorhabditis elegans* revealed the efficient cytosolic delivery of small molecules with a spatial resolution of single endosomes.

Another important pathway of DNA nanotechnology that opened new perspectives for bioimaging was the combination of DNA nanostructures with inorganic nanoparticles (NPs), pioneered by Mirkin and Alivisatos [115,116], and since then, impressive progress has been witnessed in this research field. Highly addressable DNA nanostructures combined with noble metal nanostructures such as gold nanopheres, gold nanorods, gold nanocages, and hollow gold/silver dendrites present an enormous potential for simultaneous molecular imaging and photothermal therapeutic effects. This convenient combination enables to develop selectively controlled plasmonic systems with dynamic optical response [117,118]. Kirshnan´s group constructed DNA icosahedra encapsulating a nanocrystal quantum dots (QD) and functionalized with single external molecular tags for targeting to three different endocytic ligands—folic acid, galectin-3, and Shiga toxin B-subunit to image the cell uptake by single particle tracking [119]. The live tracking of long duration compartment dynamics within cells was collected to study the endocytic pathways, following individual nanoparticles during the cellular uptake process (Figure 3f). 

The cellular uptake and intracellular trafficking of four distinct DNA origami barcoded with AuNPs—including small TDN (ST), a small rod (SR), a large tripod (LT) and a large rod (LR)—was also studied with high-resolution visualization at a single particle level, applying transmission electron microcopy (TEM) imaging in multiple human cancer cell lines [120]. Interestingly, the authors reported four distinct stages of LR internalization, describing an initial longitudinal aligning of the particles onto the membrane, followed by a rotation by 90° during membrane transversing, transporting to early endosomes, and finally to late endosomes and lysosomes. No AuNPs were found to escape endosomes to cytoplasm what could represent a major drawback of these structures to drug delivery (Figure 3g). They further described that larger nanostructures exhibited higher cellular uptake efficiency and their shape is also relevant for the interaction between DNA nanostructures and cell membrane. In comparative fluorescent-based study, the uptake of 11 distinct DNA origami-shapes has also shown to be dependent on nanostructure size, aspect ratio, and cell type [121]. 

### 5.2. DNA Nanostructures as Platforms for Diagnosis in Living Cells and Biological Fluids

Reliable, rapid and accurate real-time biosensors have been pursued as they can provide essential tools for clinical diagnosis and cell signaling pathways. The design of smart DNA nanostructures able to simultaneously monitor and quantify in real-time reactive molecules— especially those involved in a variety of physiological and pathological processes—has a significant importance for early diagnosis and tailored medicine and has becoming an attractive research topic in the last decades. Herein, we summarize the recent DNA-based sensors that have been conceived for living cells or in complex physiological milieu (Table 1). Among the diverse DNA nanostructures developed, the DNA tetrahedron stands out in biological and medical applications. This stable 3D structure can be modified covalently with functional moieties, and more importantly, its fast and simple assembling procedure improves the scalability of this nanostructure. Through different strategies, nanosensors created by this nanostructure have been proposed not only focusing on their direct use in living cells but also in their use as sensitive external devices for the diagnosis of biological samples. For instance, Li et al. [122] designed a DNA tetrahedron anchoring the responsive probes fluorescein and hydroethidine in the four vertexes to endow the simultaneous determination of pH and superoxide anion (O_2_^•−^), respectively, in living cells and in vivo. The anomalous production of these two species has been associated in the triggering of multiple diseases such as inflammation, neurodegenerative diseases, and cancer [123]. Confocal fluorescence images indicated that these nanoprobes allow the separate and concurrent detection of pH and O_2_^•-^ in living cells, and concomitantly, the downregulation of pH and upregulation of O_2_^•-^ were selectively discerned in an inflammation model in vivo. A similar DNA wireframe, a DNA triangular prism, was designed to quantify and monitoring adenosine triphosphate (ATP) inside living cells [124]. ATP is implied in many biological pathways and its level may provide important information regarding the diagnostic of many diseases [125,126]. The DNA triangular-prism encapsulated split aptamers labeled with donor and acceptor fluorophores was created to undergo FRET after the binding of two ATP molecules in the recognition modules. This nanoprobe displayed high stability, sensitivity, and selectivity for quantitative detection of ATP while being able to protect the cargo and efficiently internalize living cells. 

**Figure 4:**
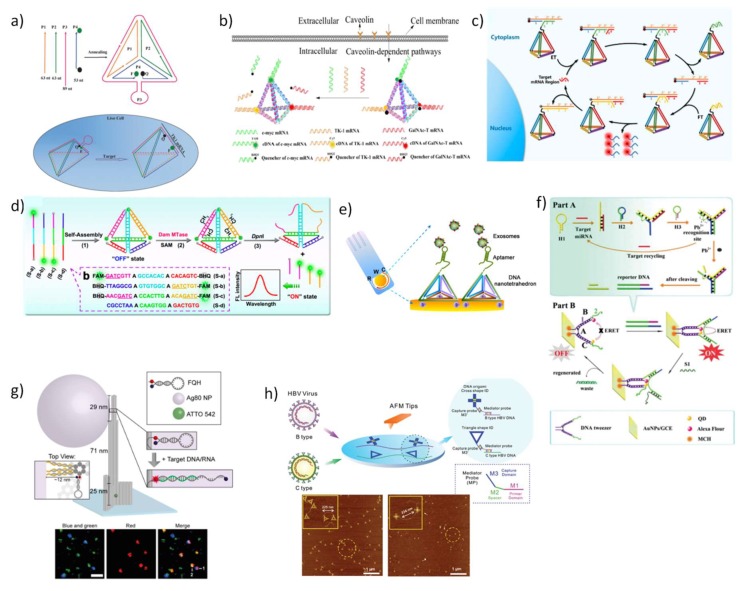
DNA nanostructures for diagnosis in living cells and biological fluids. Schematic illustration of DNA tetrahedral nanoprobes developed for detect (**a**) TK1 mRNA, adapted with permission from [131], (**b**) TK1 mRNA, GalNac-T mRNA, and C-myc mRNA simultaneously, adapted with permission from [132], (**c**) TK1 mRNA amplified by cycle strand displacement reaction, adapted with permission from [133], and (**d**) DNA methyltransferase activity, adapted with permission from [139], all designed to operate in intracellular environment and providing fluorescence readout. The immobilization of DNA nanostructures into gold surfaces have been used, for example, to detect (**e**) exosomes via redox signal, adapted with permission from [145] or (**f**) miRNA by electrochemiluminescent signal, adapted with permission from [147]. Reconfigurable DNA origami allows the detection of (**g**) Zika-specific artificial DNA and RNA, adapted with permission from [149] and (**h**) Hepatitis B genotyping [151,152] in biological samples, adapted with permission from [151,152].

Tumor-related mRNAs are important biomarkers whose expression was demonstrated to be related with tumor burden or progression, cardiovascular diseases and a vast number of other diseases [127,128]. A nature-inspired DNA TDN for intracellular mRNA detection was developed by Tay et al. [129] by conjugating a sensory molecular beacon (MB) module to one vertex of the tetrahedral structure. Accurate detection and monitoring of mRNA transcript were achieved in living cells. Other groups followed this strategy, for instance, Xie et al. [130] reported a TDN-based molecular beacon but they proposed the direct incorporation of the MB in one of the four constitutive strands of the TDN structure, as a hairpin, to increase the structural stability of the sensor (Figure 4a). Thus, when target mRNA hybridizes with the complementary sequence of the hairpin, quencher, and fluorophore are separated, leading to a strong fluorescence emission. Using TK1 mRNA as a target model, the newly designed nanosensor displayed a reliable detection of mRNA expression in living cells, and the detection limit of the fluorescence system reached a value as low as 3.2 nM. For the same purpose, He et al. [131] designed a DNA TDN nanotweezer using also FRET as signal readout mode, reaching a detection limit of 0.33 nM. Wang et al. [132] demonstrated that DNA TDN excels in detecting simultaneously three different tumor-related mRNAs in living cells. For this, three staple-strands of TDN were elongated and modified with three different fluorophores (FAM, Cy3, and Cy5) and hybridized with the recognition sequences holding the corresponding quenchers, thereby suppressing the fluorescent signal. After TDN internalization, quenching sequences are released allowing the hybridization with target mRNAs and fluorescence is restored (Figure 4b). Recently, an innovative strategy was devised involving entropy-driven signal amplification to improve the sensitivity and selectivity for a specific intracellular mRNA target inside cells. In the presence of target TK1 mRNA, the amplifier is readily initiated, triggering a cycle of strand-displacement reactions in two distinct DNA TDN, culminating in an intense fluorescence signal recovery after the separation between donor and acceptor fluorophores (Figure 4c) [133]. Ultrasensitivity was also obtained by constructing DNA pyramids that self-assemble into gold nanoparticles and lanthanide-doped upconversion nanoparticles. This doubly optically active biosensor endows exceptional plasmonic circular dichroism (CD) and luminescence detection for endogenous miRNA quantification in live cells [134]. 

Similar strategies have been used for quantify other relevant DNA or RNA biomarkers in biological fluids, such as human serum or blood [135,136,137,138]. Diao et al. developed a surface plasmon resonance sensor for detection of HIV-related DNA combining entropy-driven strand displacement and double layer DNA TDNs for signal amplification, achieving a detection limit of 48 fM and rapid diagnosis in complex biological samples [135]. Another important biomarker detected by DNA TDN-based sensor was the enzyme DNA methyltransferase (MTase) [139]. This enzyme is involved in the DNA methylation process, and if an aberrant DNA methylation occurs, it may alter gene expression resulting in tumorigenesis and tumor metastasis [140,141,142]. Experimentally, DNA TDN was assembled with three staple-strands duals labeled with fluorescein (FAM) and black hole quencher (BHQ) and a fourth staple-strand at its “OFF” state. The adenine residues present in the two edges of DNA TDN—in which the recognition sites of MTase are located—are methylated upon a methyl group transfer from the group donor of the S-adenosylmethionine (SAM) binding site to the receptor residue. These methylated sites are then recognized and cleaved by the restriction endonuclease DpNI, resulting in the collapse of the tetrahedral structure and the subsequent recovery of fluorescence signal to display an “ON” state (Figure 4d). This DNA TDN-based fluorescence biosensing system presented a limit of detection as low as 0.045 U mL^−1^ in a human serum sample [139]. The same authors proposed a tetrahedron-structured probe for electrochemical detection of methyltransferase activity obtaining a five-times higher current than captured with single-strand capture probe and higher sensibility than the fluorescent approach [143].

So far, the structured DNA TDN probes represent the large majority of biosensors developed for high-throughput analysis since highly tailorable DNA nanostructures have provided novel means to solve common problems including malfunctioning biosensor interface, multiple non-specific interactions, and low specificity of target confining. These platforms have great interest for biomarkers detection in complex biological fluids such as cell lysates, tissue extracts, and human serum. The general strategy is well defined in which three vertices of TDN are modified with thiol groups to easily anchor to a gold surface while the fourth has a functional modification disposed in the upright direction, conferring high organization to the biosensor, and consequently, improved performance [144]. The resulting electrochemical sensors have been reported to provide a sensitive method for quantitative analysis of miRNAs and DNA [137,138], reaching exceptionally low limits of detection. Also, it has been demonstrated that their valuable contribution to detecting clinically relevant protein such as pneumococcal surface protein A (PspA) peptide [145], and even cancerous exosomes (Figure 4e) [145] and cells [146]. All of these studies demonstrated that the improved sensitivity arises from the mechanical rigidity and structural stability of the 3D-pyramidal structures. In addition to DNA TDN, other nanostructures have been successful applied as biosensors in living cells. An ingenious ultra-high sensitive DNA tweezer including Alexa Fluor 488 as the donor and quantum dots (CdSe@ZnS) as acceptor bound to gold nanoparticles was proposed for the detection of miRNA inside cancer cells [147]. In a first step the target mRNAs triggers the hybridization of a second hairpin that hybridize to a third hairpin to form a Y-shaped DNAzyme, this structure is then cleaved in the presence of Pb^2+^, to originate one ds-DNA upon release of the target miRNA. Secondly, the hybridization of one strand of the amplified dsDNA to the DNA tweezer leads to the proximity of the donor and acceptor fluorophores triggering an electrochemiluminescent signal (Figure 4f). 

Taking advantage from the specificity of aptamers binding and using the HCR as amplification strategy, Song et al. [148] demonstrated the formation of highly crosslinked DNA networks able to cloak selectively cancer cells. Specifically, an anti-EpCAM aptamer recognizes epithelial cell adhesion molecule (EpCAM) and anchors the DNA initiator on the cell surface via the formation of aptamers-initiator biblocks that initiates aptamers-trigger clamped hybridization reaction (atcHCR) to form a porous hydrogel. DNA hydrogel had demonstrated high-sensitivity and specificity for entrap cancer cells and their decloaking is easily controlled by a chemical stimulus without inducing cell damage. 

The origami technique holds great potential to supply intricate platforms for diagnosis but reports of their application as biosensors in biological environments are still scarce. In this regard, a DNA origami pillar was engineered using computational tools for enable the direct detection of Zika-specific DNA and RNA in human blood serum [149]. The DNA pillar was immobilized in a surface and on its upper part a fluorescence-quenching hairpin (FQH) was attached to one extending strand for detecting the target DNA or RNA (Figure 4g). Upon binding of the target nucleic acid, the hairpin changes its initial configuration and the resulting fluorescence signal is amplified by a plasmonic fluorescent silver nanoparticle located in its vicinity. In a related study, a well characterized rectangular DNA origami incorporating twelve aptamers that specifically bind to the malaria biomarker, *Plasmodium falciparum* lactate dehydrogenase (PfLDH) was produced (Figure 4h) [150]. Resulting protein-aptamer-origami were found to be stable under human blood plasma and enable the quantification of the protein-aptamer binding through high-speed atomic force microscopy (AFM) at a detection level as low as 500 nM. Another infection with clinical interest due to its severe epidemic impact is the one caused by Hepatitis B virus (HBV). For virus infection diagnosis, different predesigned DNA origami shape ID probes, including cross and triangular nanostructures, with distinct amounts and positions of capture probes were used to identify HBV genotypes under AFM [151,152]. These two DNA-probes showed high specificity and sensitivity towards simultaneous detection of genetic variation in HBV at a single-molecule level. 

### 5.3. DNA Nanostructures as Platforms for Drug Delivery

#### 5.3.1. DNA Nanostructures for Anticancer Drugs Delivery

The use of DNA nanostructures as small-molecules delivery systems is mainly confined to the delivery of doxorubicin (DOX) because this drug is regarded as one of the most effective and widely used chemotherapeutic drug approved by FDA. Moreover, the planar aglycone moiety of this anthracycline can intercalate between base pairs of DNA facilitating drug loading. This feature was originally considered to be the mechanism for cytotoxicity, but currently it is known that in addition DOX inhibits topoisomerase II [153] and this latter mechanism seems to be the primary source of cytotoxicity. Besides this, anthracyclines hamper nuclear helicases to unwind duplex DNA during the process of strand separation [154] and also can undergo reduction leading to the formation of reactive compounds that can damage lipid membranes [155]. All these events contribute for a potent but non-specific cell killing which limits DOX therapeutic dose due to the unwanted side effects in non-tumoral cells [156,157]. Furthermore, DOX has been shown to provoke drug resistance in cancer cells, detected either in research experiments or in clinical studies [158,159]. Due to this, it is considered urgent to create a vehicle intended to internalize exclusively cancer cells and circumvent cell resistance. 

**Figure 5:**
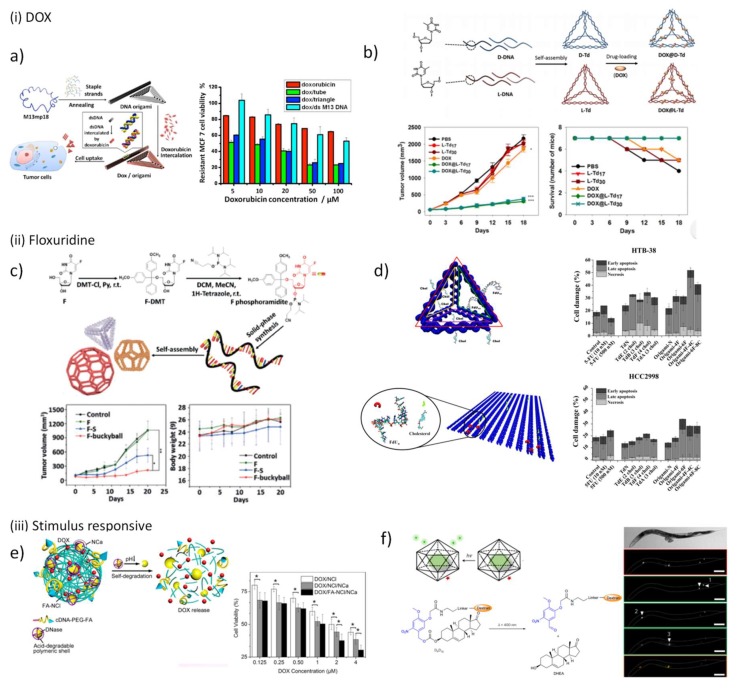
Examples of DNA nanostructures developed for the delivery of small molecules. (**a**) Anti-neoplastic DOX-loaded DNA triangles and nanotubes for circumvent multidrug resistance, adapted with permission from [162]. (**b**) Targeted antitumor treatment by the DOX loaded to chemically modified L-TDNs (DOX@L-Tds), adapted with permission from [165]. (**c**) In vivo evaluation of antitumor effect using F-buckyballs with floxuridine (F), adapted with permission from [170]. (**d**) Evaluation of the apoptotic effect promoted by DNA TDN and DNA origami integrating 5-fluoro-2’-deoxyuridine (FdU) oligomers and cholesterol moieties, adapted with permission from [171]. (**e**) Self-degradable DNA nanoclew sensitive to pH changes for the delivery of DOX, adapted with permission from [179]. (**f**) DNA octahedron with precise delivery of cargo upon photoirradiation in *C. elegans*, adapted with permission from [114].

Self-assembled DNA nanoscaffolds are ideal candidates to intercalate this drug within DNA base pairs and accomplish its delivery, and recently have been widely explored for this purpose (Table 2). With this in mind, Huang and co-workers designed an aptamer-decorated DNA icosahedron with DOX showing an efficient and specific cytotoxic action against epithelial cancer cells [160]. In 2012, Högberg and coworkers [161] taking advantage from the large number of available positions to intercalate DOX tested in vitro the feasibility of two DNA nanostructures, a straight nanotube (S-Nano) and a twisted nanotube (T-Nano), designed with caDNAno [50] as delivery systems to human breast cell lines. The authors observed an efficient delivery of DOX, with a release controllable by the level of twist imposed to T-Nano, being the nanotube twisted over 12 bp/turn the most promising nanocarrier. In the same year, Ding and coworkers employed 2D and 3D DNA nanostructures loaded with a high concentration of non-covalently attached DOX to demonstrate the ability of these vehicles to overcome cell resistance. Both in doxorubicin-nonresistant and doxorubicin-resistant cancer cells, the constructed DNA nanocarriers exhibited a prominent cytotoxicity (Figure 5a) [162]. The same evidence was obtained by Castro and coworkers when employing a rod-like origami to deliver another anthracycline drug, the daunorubicin, in a leukemia model [163]. In the follow up project, Ding and coworkers proved in mice that DNA nanostructures excels to deliver chemotherapeutic drugs to tumors [164]. Surprisingly, they also found a correlation between drug uptake efficiency and the configuration of DNA origami, being triangular-shaped DNA origami more efficient than the related rectangle and tube scaffolds. The full modification of TDN DNA strands by substituting the natural d-sugar DNA (d-TDNs) by mirrored l-sugar DNA (l-TDNs) was carried out to overcome the intrinsic instability in vivo of these simple wireframe nanoscaffolds (Figure 5b) [165]. Both improved serum stability and enhanced cellular uptake were observed for l-TDNs-DOX, and after systemic injection, a high tumor-specific accumulation while minimizing cytotoxicity at non-target organs was registered, followed by prolonged in vivo residence and improved DOX potency. These in vitro and in vivo findings provided evidence of DNA origami as promising anticancer drug delivery systems and prompted the study of other classes of chemotherapeutic agents.

Sleiman and coworkers [166] validated a new strategy for the delivery of BKM120, a pyrimidine-derived selective PI3K inhibitor approved by FDA as an anticancer drug for the treatment of chronic lymphocytic leukemia (CLL) [167]. Spherical nucleic acids (SNAs) are composed by a hydrophilic DNA shell and hydrophobic core which can accommodate lipophilic drugs and they have become particularly relevant in drug delivery field [168,169]. In detail, the proposed SNAs are generated by the assembling of DNA-polymer conjugates (HE_12_-DNA), consisting of a 19-mer DNA sequence linked to 12 dodecane (hexaethylene, HE) and BKM120 were entrapped in the resulting hydrophobic core. BKM120-loaded SNAs induced apoptosis in primary patient CLL lymphocytes and acted synergistically when co-delivered with DOX. In vivo assays in mice demonstrated promising results, evidencing full body distribution, long circulation times, and high accumulation in tumors. 

Recently, DNA polyhedra were designed to hide in their scaffold floxuridine-integrated DNA strands, synthesized and self-assembled into DNA nanostructure with a precise drug loading cargo (Figure 5c) [170]. These nanostructures were reported to improve the pharmacokinetics of free drug in vivo and inhibited the proliferation of tumor cells in vivo, especially when the bulky ball nanostructure is used. Following the same purpose, we recently proposed the conjugation of 5-fluoro-2’-deoxyuridine oligomers (FdUn) in two DNA nanostructures, a DNA TDN and a rectangle DNA origami for colorectal cancer treatment (Figure 5d) [171]. To enhance cell internalization, cholesterol moieties were inserted in the 5’-end of some inherent nanostructure staples. The nucleotide 5-fluoro-2’-deoxyuridine monophosphate is one of the products of 5-fluorouracil (5-FU) intracellular conversion and is responsible for inducing “thymineless cell death” [172,173]. The effect of 5-fluoro-2’-deoxyuridine oligomers (FdU_n_) have been inspected in other studies and showed higher efficiency to trigger cell death than the parent drug 5-FU which is currently used clinically in colorectal cancer treatment [174,175,176]. These nanostructures were successfully validated as a new type of FdU delivery vectors and have demonstrated to overcome 5-FU cell resistance. The cholesterol content showed to be positively correlated with the cytotoxic effect of the nanostructures. In comparison, both DNA nanostructures attained comparable cytotoxic effect however TDN has a higher antiproliferative action, since its concentration is higher than DNA origami.

DNA-based stimuli-responsive drug delivery systems have been also exploited recently due to the obvious need to precisely release drugs in a specific cells or tissues, decrease systemic toxicity and avoid under- and over-dosing. DNA nanostructures formed by RCA methods have been successfully applied to increase the payload of DOX [177,178]. Using the same methodology, a degradable DNA nanoclew (NCl) was created upon assembly of long-chain ssDNA synthesized by RCA containing repeated GC-pairs to allocate DOX along with folic acid conjugates and embedded acid-responsive DNase I nanocapsules (NCa) (Figure 5e) [179]. When this multifunctional nanostructure internalizes a cancer cell through endocytosis mediated by folate receptor and faces the acidic conditions in endolysosome, the activity of DNase I is maximized upon degradation of the polymeric cover of NCa, which in turn triggers the self-degradation of NCl and consequently the release of DOX. In vitro cytotoxic studies validated the efficiency of the multifunctional system DOX/FA-NCl/NCa exhibiting a half-maximal inhibitory concentration (IC_50_) of 0.9 µM while DOX/NCl has an IC_50_ of 2.3 µM. As mentioned in the previous section, DNA icosahedron was used by Kirshnan and co-workers to demonstrate the uptake of DNA nanostructures in living cells. Lately, these same nanostructures were employed to site-specifically release a neurosteroid drug, the dehydroepiandrosterone (DHEA), upon photoirradiation endowing spatial and temporal control with single-endosome precision in *C. elegans* (Figure 5f) [114]. 

#### 5.3.2. DNA Nanostructures for Therapeutic Oligonucleotides 

Therapeutic oligonucleotides (ODNs) such as short-interfering RNA (siRNA), antisense oligonucleotides (ASOs), microRNAs (miRNAs), synthetic mRNAs, and CRISPR-CAs9, are able to target undruggable genes following different mechanisms with high selectivity, enabling the treatment of any disease-related gene [180,181]. Recently, FDA approved several oligonucleotide-based drugs [182] and, in 2018 the first drug based on RNA interference, Partisiran (Anylam Pharmaceuticals), sparking researchers’ interest in this field after a two-decade wait. Apart from the gene-silencing ODNs, a new class of synthetic therapeutic ODN containing unmethylated cytosine phosphate guanine (CpG) motifs became popular for immune stimulation in cancer immunotherapy [183]. This CpG ODN is specifically directed to act as immunostimulant upon recognition by Toll-like Receptor 9 (TLR9) expressed in antigen-presenting cells (APCs) such as dendritic cells and macrophages [184]. 

Since their discovery, ODN chemistries have evolved to increase stability, avoid innate immune responses, and increase potency while lowering off-target activity profiles [185,186]. However, the delivery of nucleic acids to cells is hurdled and represents still a major challenge for their clinical translation as therapeutics. The effective cellular delivery of molecules with therapeutic value is more successful in the case of small molecules, however, in what regards nucleic acids, the hostile cellular environment rapidly degrades and gets rid of these charged molecules if they are not properly escorted into the cells. DNA nanostructures brought up new platforms for developing dynamic and responsive vehicles able to integrate DNA and RNA-based therapeutics simply by hybridization, protect them in extra- and intracellular media and circumvent biological barriers during cell entry. Examples of DNA-based nanostructures developed for deliver therapeutic oligonucleotides are listed in Table 2.

In 2012, Anderson and coworkers [187] reported for the first time the behavior of DNA nanostructures in mice using a DNA TDN decorated with up to six small interfering RNA (siRNA). Therapeutic cargo and tumor targeting agents were attached to the nanostructure via hybridization with a precise location and orientation. Folic acid (FA) conjugated to DNA tiles was the most efficient targeting agent among the ones tested, with its gene silencing activity being dependent on both number and spatial orientation. In vivo fluorescence molecular tomography images evidenced a high accumulation of the nanostructures in the tumor and kidney at 24 h post-injection, corroborating well the biodistribution found in experiments ex vivo at 12 h post-injection. In addition, the chemical modification of siRNA with 2’-*O*-methyl nucleosides increased serum stability and reduced the potential of immune stimulation. Almost simultaneously, Liu et al. [188] showed that DNA TDN excel in assembling a model antigen and CpG adjuvants in a controllable three-dimensional configuration and induce a strong, specific, and long-lasting antibody response in immunized mice. This work was the first evidence that DNA nanostructures serve as excellent platforms for the construction of vaccines. Furthermore, Liedl and Rehberg groups demonstrated that CpG-decorated DNA nanotube microinjected in the skeletal muscles of mice are efficient to trigger immunogenic responses [189].

**Figure 6:**
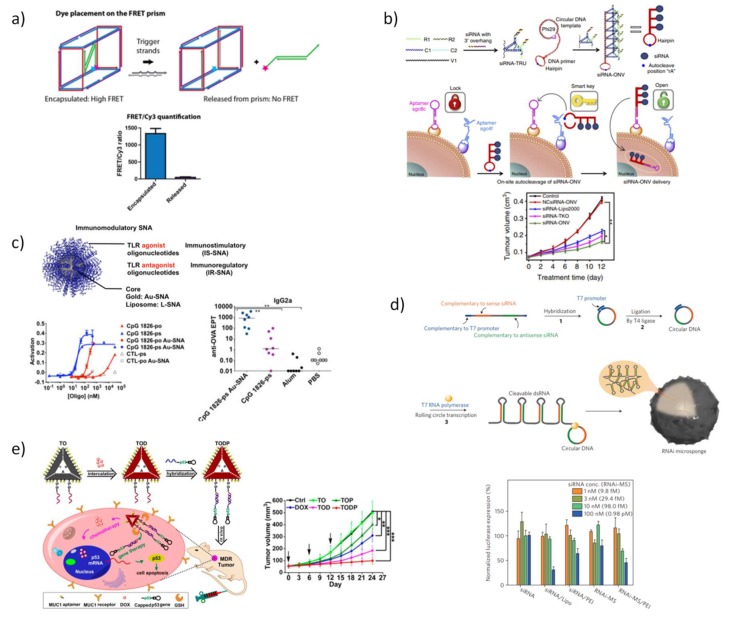
Examples of DNA nanostructures developed for the delivery of therapeutic oligonucleotides. (**a**) Release of siRNA encapsulated in DNA prisms monitored by a FRET reporter system, adapted with permission from [191]. (**b**) Schematic illustration of “dual lock-and-key” DNA-based nanovehicle for a cell-specific siRNA delivery, adapted with permission from [192]. (**c**) Immunostimulatory-SNAs exhibited high potency and high IgG2a serum titers, adapted with permission from [195]. (**d**) Process of rolling circle transcription (RCT) for the assembling of RNAi microsponges, where a linear ssDNA including antisense and sense of anti-luciferase siRNA is hybridized with DNA strands holding the promoter T7 promoter sequence, adapted with permission from [196]. (**e**) DNA origami-based device for synergistic breast cancer treatment using RNAi therapy (p53 gene) and chemotherapy (DOX), adapted with permission from [203].

This convenient combination of DNA nanostructures with therapeutic ODNs was also investigated by Sleiman and co-workers [190] that designed, synthesized and characterized a simple and economic DNA-based triangular prism composed with only three DNA strands of 92–96 bases in length, possessing regions for site-specific hybridization able to brace up to six antisense oligonucleotide strands. The assembly of antisense DNA in the 3D-DNA scaffold significantly increased the half-life of the therapeutic cargo in almost four-fold while holding four and six antisense units. In mammalian cells, the antisense prism displayed better gene silencing than antisense alone, more specifically, prisms holding four and six antisense strands were able to maintain gene knockdown up to 72 h. Later in 2016, the same authors rationally designed a more robust trigger-responsive DNA prism for encapsulate, protect, and selectively release a siRNA (Figure 6a) [191]. Two trigger antisense oligonucleotide strands that specifically recognize the apoptotic genes, Bcl-2 and Bcl-xL were used to control siRNA release and each were located in opposite faces of the prism to reduce the number of potential misassembled structures. To protect single-stranded portions from biological harms, the endogenous phosphodiester backbone was replaced by phosphorothioate. In addition, a full optimization of the structure was achieved by inserting locked nucleic acids monomers to maintain the tridimensional arrangement even in a reduced salt concentration and under elevated temperature conditions; while the insertion of hexaethylene glycol units further increased the propensity to adopt single stable structures. The carefully designed DNA prism was found to efficiently release siRNA in a cellular environment and induced gene knockdown in mammalian cells. Also for control, the precise release of siRNA in intracellular environment, a “dual lock-and-key” DNA-based nanovehicle was devised, consisting in an auto-cleavable siRNA–loaded hairpin structure that acts as a “smart key” to trigger cell siRNA internalization upon hybridization in a serial manner with two kinds of aptamers, sgc8c and sgc4f (Figure 6b) [192]. This strategy provided increasing delivery cell specificity, reduced off-target cytotoxicity over the single-receptor delivery systems and demonstrated to be efficient in therapeutic applications via target VEGF gene silencing. Using a different approach, Jensen et al. [193,194] proposed a new array in which siRNA duplexes were covalently bound to functionalized gold nanoparticles forming densely packed spherical nanostructures. These SNAs were tested preclinically and demonstrated an excellent ability to cross in vivo the blood brain barrier (BBB) and high efficacy on reducing oncogene expression in severe glioblastoma multiforme. This strategy is currently undergoing clinical trials (NU-0129) in early phase I while other therapeutic oligonucleotides have been exploited for other diseases [169]. The SNAs have been also used for stimulating or regulating immune responses by carrying agonizing and antagonizing endosomal toll-like receptors (TLRs) (Figure 6c) [195]. Immunostimulatory-SNAs exhibited up to approx. 80-fold higher potency than unformulated CpG oligonucleotides, 700-fold higher antibody titer, 400-fold higher cellular responses to a model antigen and high efficiency in the treatment of mice lymphomas. For immunoregulatory-SNAs, the potency was also increased up to eight-fold and a reduction of 30% in fibrosis score in mice with nonalcoloholic steathepatitis, demonstrating the attractive potential of SNAs for boosting immunotherapies. SNAs have revealed great efficiency and versatility as therapeutic platforms developing the fields of diagnostics, gene regulation and immunotherapy [169]. 

Furthermore, techniques such as rolling circle transcription (RCT) or rolling circle amplification (RCA), both derived from natural rolling circle replication (RCR), have been used to increase the ODN therapeutics payload. These methodologies generate large ss-DNA or ss-RNA which self-assemble to form globular structures or “nanoflowers” [196] or else remain unfolded to form free-standing RNA membranes [197], and in some cases aptamers sequences may be also included to facilitate cell targeting [198]. The delivery of long polymers of siRNA that self-assembles into nanoscale pleated sheets of hairpin RNA forming sponge-like microspheres exponentially increased the siRNA payload capacity (Figure 6d) [196]. In average, each nanosphere contains approximately 102,000 siRNA copies which are fragmented and released inside cells. The addition of the cationic agent, polyethylenimine (PEI) helps to further compact the microspheres and allows achieving a significant gene silencing efficiency at siRNA concentration as low as 2.1 fmol [196]. DNA or RNA-based stimuli-responsive nanoparticles coated with a glutathione-sensitive chitosan polymer were also designed to release multiple RNA copies exclusively inside cells by the action of cellular ribonuclease RNase H [199]. Empowered by the structure programmability of the nucleic acids, the synthesis of multiple components of polymeric siRNA was performed to enable simultaneous silencing of target genes [200]. Also based on these methodologies, a new cancer immunotherapy agent to prevent postsurgical tumor relapse was created, by constructing a delivery carrier for the controlled release of CpG oligodeoxynucleotides and anti-PD-1 antibody (aPD1) to trigger the immune response against cancer cells [201]. The combination of these two immunostimulatory agents act synergistically at tumor site, displaying a significant regression in tumor growth, with approximately 40% of mice surviving 60 days. In a recent work, Zhu et al. [202] developed a new methodology to generate hybrid DNA-RNA nanostructures (iDR-NCs) through the combination of RCR and RCT in the same reaction system, to respectively construct CpG and Stat3-silencing shRNA chains, to promote immunostimulation synergistically by triggering TLR9 and STAT3 signaling pathways (Figure 6d). Biocompatible PEG-grafted polypeptide copolymers were synthesized and used to further condense iDR-NCs. These copolymers enhanced their delivery efficiency, increased biocompatibility and enabled the attachment of peptide neoantigens in nanoparticles surface via hydrophobic interactions. Remarkably, iDR-NC-neoantigen nanostructures presented higher potential for inducing T cell memory than CpG and also inhibited the progression of neoantigen-specific colorectal tumors, opening new perspectives to the development of triple-co-delivery nanocarriers to be applied in immunotherapy. 

Interestingly, recent studies exploited the high addressability of DNA nanostructures to co-deliver therapeutic oligonucleotides and anticancer drugs. A triangle DNA nanostructure was tailored to load an important tumor suppressor gene, the p53 gene, and DOX for combining therapy of multidrug resistant breast tumor (MCF-7R) (Figure 6e) [203]. Having both MUC1 aptamers for targeted delivery and glutathione reduction responsiveness for effective release of genetic cargo, these triangular DNA promoted a strong reduction of tumor growth both in vitro and in vivo without apparent systemic toxicity. The same group reported a concurrent strategy to fabricate both RNAi and chemodrugs loaded multifunctional DNA nanoscaffolds to act synergistically in the treatment of multidrug-resistant tumors in vivo [204]. The DOX pre-loaded triangular DNA origami hold now two linear small hairpin RNA (shRNA) transcription templates for silencing two MDR-associated genes, the gene of P-glycoprotein (shPgp), a typical drug efflux pump and the gene of surviving (shSur), an anti-apoptotic protein. As demonstrated in previous reports, triangular DNA origami with shPgp alone do not induce significant tumor burden [205,206] and it was corroborated again in this work, and the same was detected for DOX alone which is not active in multidrug resistant tumors. The multifunctional triangular DNA exhibited the greatest antitumor effect in mice supported by a potent silencing effect of the shPgp and shSur genes. 

#### 5.3.3. DNA Nanostructures for Therapeutic Proteins Delivery

Proteins are responsible for executing central cellular tasks, including gene replication and regulation, signal transduction, stimuli response, metabolic catalysis, and mediation of the transport of molecules between inner and outer cellular compartments. The fast growth of DNA nanotechnology in the past decades enabled the programmable combination of important protein therapeutics, such as important enzymes, antibodies, cytokines or transcription factors, with nucleic acids making use of their sequential codes and specific interactions as key factors for DNA-protein assembly [207,208,209]. To date, a reasonable number of DNA nanostructures have been studied to immobilize and confine [79,86,210,211,212] protein therapeutics in space and time to enhance their stability, control and regulate their function, and facilitate the study of their structural dynamics at the single-molecule level. These fundamental works provided a solid foundation to the development of tailored and efficient protein therapeutics delivery DNA nanostructures (Table 2). 

**Figure 7:**
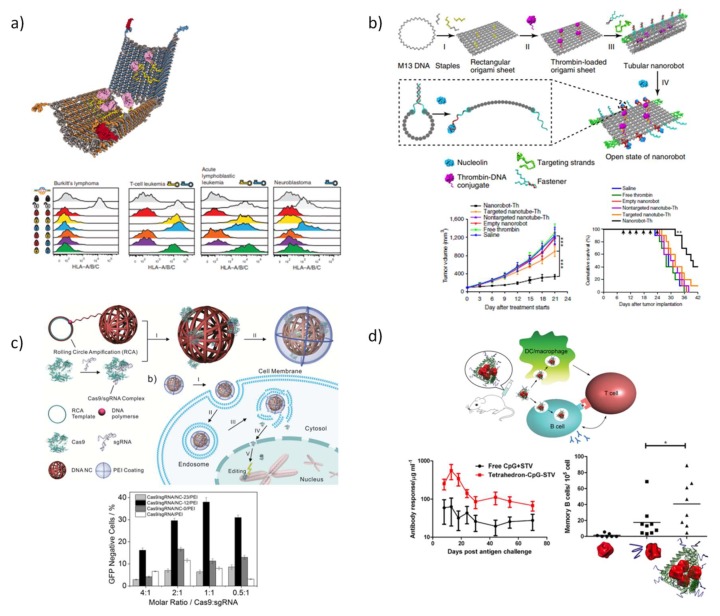
Examples of DNA nanostructures developed for the delivery of therapeutic proteins. (**a**) Schematic view of logic-gated nanorobot with protein payloads tested in different cell lines, adapted with permission from [81]. (**b**) Thrombin-functionalized DNA rectangle nanorobot (nanorobot-Th) incorporating functional fasteners and targeting strands administered in MDA-MB-231 tumor-bearing mice, adapted with permission from [214]. (**c**) Schematic representation DNA nanoclews designed for CRISPR-Cas9 delivery, adapted with permission from [215]. (**d**) Design of DNA adjuvant-antigen vaccine holding CpG ODN adjuvant molecules (purple ribbons) and model antigen streptavidin (red surfaces). The profiles for anti-STV IgG level after antigen administration and the specific memory B cell response in mice are also shown, adapted with permission from [188].

Church and co-workers [81] created a hexagonal barrel for transport a combination of antibodies, composed by two domains which are covalently linked by single-stranded scaffolds hinges in the rear, while the front of the device is non-covalently fastened by staples modified with DNA aptamers-based locks (Figure 7a). A pairwise combination of three aptamers, 41t, TE17, and sgc8c was employed to obtain a selective regulation of the nanorobot function. When in the vicinity of the aptamer targets, the fastener duplexes detach, acting as ‘entropic springs’ that provide the mechanical forces required for opening the barrel. Loading the nanorobot with a combination of human CD33 and antibody to human CDw328 Fab´ fragments and using a pair of 41t locks, an arrest in the growth of the leukemia cell (NKL) was detected in a dose-dependent fashion. Likewise, robots loaded with antibody to human CD3ε Fab´ and antibody to flagelin Fab´ successfully enhanced T-cell activation. Later in 2014, Amir et al. [213] employed the same DNA devices emulating sophisticated logic gates that can sense the presence or absence of their cognate protein cues and trigger the device opening to release the cargo. This work was the first evidence that this strategy successfully enables performing logical and dynamic operations in living animals.

Autonomous DNA nanocarriers for proteins were recently proposed by Li et al. [214]. In this work, the authors designed a nanorobot to specifically deliver the therapeutic protein, thrombin, exclusively into cancer cells by functionalizing the conventional rectangular origami with different strands that act as fasteners and targeting to mediate delivery (Figure 7b). DNA aptamers, AS1411, incorporated in origami trimmings were meant to close the origami sheet into a tubular shape for shielding thrombin attached to the inner surface, through hybridization with DNA complementary strands, and expose the payload in response to the presence of nucleolin. Twenty-four hours after administration of DNA nanorobot-Th into breast tumor xenografted mice, the vessels in tumor region were found to be efficiently occluded, and by 72 h, dense thrombosis in all tumor vessels was detected. A considerable reduction of tumor growth was also reported in poorly vascularized ovarian cancer cells and particularly effective in melanoma mouse model. In this latter model, the nanostructure was demonstrated to affect not only the primary tumor but also avoided the formation of metastasis. With the assistance of cell-targeting aptamers, nanorobot-Th was demonstrated to be harmful only for tumor-associated blood vessels and safe in the normal tissues of both mice and normal Bama miniature pigs.

The yarn-like DNA nanoclews (NC) have been recently exploited for the targeted delivery of protein based therapeutics, like the CRISPR-Cas9 system [215] or the cytokine [216], deemed tumor necrosis factor-related apoptosis-inducing ligand (TRAIL). These DNA NC are synthesized by rolling circle amplification (RCA), where a short DNA is amplified to generate a long ss-DNA using a circular DNA template and DNA polymerases, that through base-pairing drive spontaneously to their self-assembly into spherical cages. Sun et al. [215] taking advantage from the programmability of DNA-based nanostructures, fabricated NCs capable to incorporate simultaneously the molecules required for genome editing, the Cas9 protein and single guide RNA (sgRNA) (Figure 7c). An electrostatic coating of NCs was performed by adding the well-characterized cationic polymer polyethylenimine (PEI, 40 KDa), in order to reverse the charge of Cas9/sgRNA/NC complex and concurrently increase their ability to escape the endosome. Results revealed that after 6 h, fluorescent labeled Cas protein was found inside the nuclei of human bone osteosarcoma epithelial cells (U2OS), being Cas9/sgRNA/NC/PEI mainly internalized through lipid rafts and macropinocytosis. Genome editing was successfully confirmed by in vitro and in vivo methodologies while preserving cell viability. The partial complementary between DNA NCs and sgRNA guide sequence benefits Cas9-driven genome editing, allowing the establishment of a design rule for enhancing Cas9/sgRNA complex delivery. Given the utility of CRISPR-Cas9 as a gene-editing tool, if the problems associated with its off-target rate and delivery efficiency can be overcome, new perspectives may be opened for biocomputing.

In a different approach, the same authors [216] proposed the decoration of two DNA NC encoding complementary sequences with cytokines protected by a liposome shell degradable by phospholipase A2 (PLA2), which is an enzyme commonly overexpressed in tumor microenvironment. The model cytokine TRAIL was attached to NCs tridimensional backbone through Ni^2+^-polyhistidine affinity, assuming that maleimide activated nitrilotriacetic acid was previous conjugated to the NCs for chelating Ni^2+^. NCs alone presented a mean hydrodynamic size of ca. 100 nm and a negative net charge (ZP = −21 mV) thereby the encapsulation of these structures with the degradable liposome, increased the mean hydrodynamic size to ca. 215 nm, maintaining the negative net charge. When both encapsulated NCs were mixed in the presence of PLA2, the formation of micro-scaled fibers was observed, resulting from the hybridization of the complementary constitutive strands of NCS. In human colorectal carcinoma cells, COLO 205 cell line, separately NCs were internalized into cells via clathrin and lipid raft mediated pathways, while co-administered NCs treated with PLA2 mostly bound extracellularly to the death receptors of cell membrane, thus boosting the apoptotic signaling.

Besides cancer cell uptake, immunostimulation responses were also investigated using DNA TDN to anchor the complex formed between a model antigen, streptavidin (STV) and an adjuvant, the CpG oligonucleotides (CpG-ODN) to enhance the activation of immune cells (Figure 7d) [188]. Fully loaded Td (Td-STV-CpG ODN) is readily internalized in antigen-presenting such as like mouse macrophage-like cells (RAW 294.7) and primary dendritic cells, also observed in other studies using different Td architectures and cell lines. The antigen-adjuvant co-assembly has shown to be relatively safe, because no anti-dsDNA antibodies against the tetrahedral-shaped structure were detected in the mouse serum after 18 days post secondary immunization. Experiments in immunized mice demonstrated the development of a stronger long-term immunity against antigen when mice were treated with Td-STV-CpG ODN than the free administration of CpG-STV. 

#### 5.3.4. DNA Nanostructures for Chemotherapy Combined with Phototherapy

Photodynamic therapy (PDT) is a FDA-approved non-invasive therapy for cancer and several non-malignant diseases so as infections and inflammatory conditions, by exploiting the vulnerability of cells against reactive oxygen species (ROS) [217,218,219]. PDT consists on the administration of a photoactivable fluorophore, termed photosensitizer, into the target cells or tissues that are activated through absorption of visible light to trigger a cascade of reactions resulting in an apoptotic or necrotic response, with minimum systemic toxicity. Nevertheless, the application of PDT for the treatment of deeper tissues is still restricted by a large attenuation in potency as light goes through deeper tissues and due to the low concentration of oxygen in the inner tissue microenvironment. Photothermal therapy (PTT) appeared as an alternative technique to irradiate deeper tissues since it applies near-infrared light (NIR) [220,221,222]. NIR absorbing materials converts the light into heat radiation and the effect of this thermal energy culminates in tissue ablation. Some concerns have been raised regarding cytotoxicity and efforts were made to mitigate it, either by reducing the laser power during ablation treatments, or by reducing indiscriminate necrosis in non-target cells, or by reducing the cytotoxicity of photothermal materials [220,223]. Importantly, the solution to mitigate some of the side effects of these two approaches and to increase their therapeutic outcome relies in the use of multifunctional nanomachines that can combine photodynamic or photothermal effect and chemotherapeutic effect in a cooperative manner. In addition, the optical properties of the light absorbing materials can further provide molecular imaging, highly sought in theranostics. Thereby, advances in DNA nanotechnology contributed to construct smart theranostics nanodevices.

**Figure 8:**
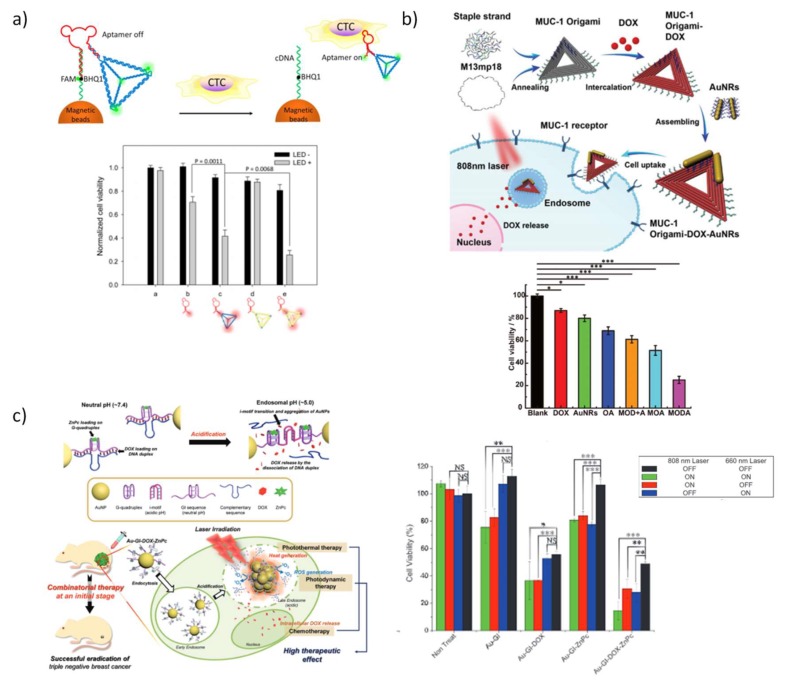
Examples of DNA nanostructures for chemotherapy combined with phototherapy. (**a**) “Sense and Treat” DNA nanodevices incorporating DOX and photosensitizers, depicted as green dots, for synergetic removal of circulating cancer cells. Adapted with permission from [224]. (**b**) Schematic illustration of the strategy followed by Ding and coworkers, [228] using a triangle DNA origami (MODA) rationally modified with MUC-1 aptamer strands (green), capture strands (blue), gold nanorods (NR), and DOX added through intercalation with DNA base pairs. Adapted with permission from [228]. (**c**) Schematic representation of Au-GI nanomachine providing triple combination of photothermal, photodynamic and chemotherapy, adapted with permission from [230].

For example, a “sense-and-treat” strategy intended to specifically eliminate circulating tumor cells (CTCs) by combining chemo- and photodynamic therapies (Figure 8a) [224]. Such DNA nanostructures involve the deposition of a DNA-based system on magnetic beads that are able to sense CTCs through aptamers-guided interaction carrying itself a DOX-loaded DNA TDN labeled with a photosensitizer. The dynamic nature of ‘sense-and-treat’ device plays a major role in the construction of a sensitive and robust molecular sensing while inducing specific cell death. Another multifunctional diagnosis-therapy integrative system was built to co-deliver DOX and the photosensitzer 5, 10, 15, 20-tetrakis (1-methylpyridinium-4-yl) porphyrin (TMPyP4) embedded on magnetic RNA nanoflowers that selectively senses cancer cells by detection of folate receptors [225]. As probes, these RNA nanoflowers revealed a limit of detection as low as 50 HeLa cells while in vivo studies confirmed the potent synergy obtained from the combination of DOX and photodynamic therapy. Meanwhile, a smart network using aptamer-based logic gates was created to autonomously and more precisely distinguish cells in mixed populations [226]. In this implementation, the selected aptamers bind specifically to two or three cell-surface proteins, thus leading to toehold-mediated strand displacement reactions that report the diagnosis on modular AND, OR, and NOT Boolean logic gates. Once having pinpointed the target cell type, the DNA-based logic devices produce a therapeutic effect by activating a photodynamic response to eliminate the malignant cell. 

Ding and coworkers fabricated a set of dual-functional DNA origami with gold nanorods (AuNR) to afford both two-photon bioluminescence and photothermal ablation in tumor cells and in a tumor-bearing mice model [205,227,228]. In the initial work, the authors unveiled that triangle shaped DNA origami exhibits higher cellular accumulation, enhanced antitumor efficacy, two-photon cell imaging and photothermal effect than bare shaped DNA origami [227]. This latter cannot provide imaging or therapy function directly. In fact, the triangle shaped DNA-origami-AuNR were then used to achieve in vivo cancer imaging and photothermal therapy simultaneously [205]. As probes, these platforms can improve imaging quality and decreased dose. As therapeutics, they responded to NIR irradiation and effectively inhibited tumor reformation and prolonged the survival of unhealthy mice. The survival rate of mice was 80% after 30 days post-treatment, which largely differs from the almost null survival rate of untreated mice. In a follow up work, DOX, the aptamers MUC-1 and AuNRs were incorporated in triangle shape DNA origami templates for circumvent the multidrug resistance of mucin protein overexpressed (MCF-7/ADR) cells through a targeted chemo-thermal therapeutic system (Figure 8b) [228]. These complete nanotheranostic platforms acted synergistically to overcome cell resistance, with NIR irradiation being responsible for the downregulation of the P-glycoprotein (multidrug resistance pump). To reinforce these results, another targeted chemo-thermal therapeutic system confirmed this successful combination in tumor-bearing mice [229]. The combinatorial chemo, photodynamic and photothermal therapeutic effects were included in a single DNA-decorated Au nanomachine (Figure 8c) [230]. In this study, a dual function DNA sequence containing i-motif and G-quadruplex structures, called GI sequence, is linked to functionalized small-sized gold nanoparticles (15 nm) and a complementary strand ensures the intercalation of DOX. G-quadruplex structure carries a hydrophobic synthesizer, the zinc phtalocyanine (ZnPc), while the i-motif structure is pH-responsive. After being internalized and exposed to acidic pH, a transition of i-motif structure takes place leading to a pH-responsive release of DOX in target cells, and subsequently, the i-motif formation between Au-GIs that culminates in AuNPs aggregation for a photothermal effect upon NIR irradiation (808 nm). Ultimately, the illumination by 660 nm light induces the activation of ZnPC for photodynamic therapy. Confocal laser scanning microscopy monitored the pH-responsive release of DOX inside endosomes. When applied the Au-GI-DOX-ZnPc nanomachines and subjected the tumor to 660 and 808 nm dual-light irradiation, a strong reduction and sustained tumor growth inhibition was detected.

Altogether, the aforementioned case studies demonstrate the great potential of the combined usage of programmable functional DNA nanostructures with inorganic nanoparticles “as smart molecular doctors”, enabling not only efficient treatment but also real-time bioimaging and high-throughput diagnosis.

## 6. Conclusions 

DNA-based nanotechnology has made remarkable advances towards in-cell and in-vivo applications holding a great potential to revolutionize the field of synthetic biology. Herein, we summarized the current leading strategies used to build DNA nanostructures, including the tile-based, origami-based, nanoparticle-conjugated, rolling circle amplification (RCA), and the hybridized assembly approaches with application in biomedical scenario. Scaffolds ranging from the simple tetrahedron to intricate origami have been exploited as dynamic platforms for incorporate therapeutic, targeting molecules, and organic and inorganic fluorescent probes. DNA nanotechnology offers not only a precise control over the structures’ size, shape and surface functionality but also provides modular assembling techniques for design external-stimuli responsive devices suitable for sensing, computing and diagnosing. In addition, the large spectrum of the oligonucleotide chemistries can further provide tools to enhance structural integrity, cell internalization and cell specificity for improving their use in in vivo drug delivery, facilitate cell imaging and offer disease diagnosis. In this context, new methodologies have been developed to increase therapeutics loading capacity and amplify the fluorescence signal of diagnostic devices. Targeted DNA structures anchoring a precise number of functional moieties that specifically recognize cell receptors, such as antibodies, peptides and aptamers, have made progresses towards the goal of enhance cell delivery and are currently inserted in competitive vehicles as a strategy to overcome cell hurdles. DNA multifunctional nanomachine combined with photodynamic and photothermal therapies represent a powerful anti-cancer therapy by attacking the cancer in a diverse and cooperative manner to attain a desirable therapeutic outcome.

Despite all these attractive characteristics, there is also a limiting bottleneck that hampers the use of DNA nanostructures in a larger number of animal experiments. DNA is more expensive than the conventional polymers used in the nanotechnology field, which potentially difficult DNA origami scale-up and limits to enlarge the scope of DNA nanotechnology. In DNA origami production, shorter strands are produced through costly and time-consuming chemical synthesis, allowing just small amounts of material. New solutions have been recently exploited to circumvent this major drawback. Dietz and co-workers employed bacteriophages to produce both the scaffold ss-DNA and the short staple strands while drastically reducing the price of production [231], which might open up new perspectives to scale up DNA origami technology. DNA nanotechnology is still in an early stage but represents today a forefront frontier for the biomedical field, offering great perspectives for the development of theranostics which are considered a research-demanding area to foster predictive, preventive, and personalized medicine.

## Figures and Tables

**Table 1:** DNA-based nanosensors designed for the detection of biomarkers in biological fluids and in living cells

DNA Nanostructure	Biomarker	Detection Limit	Signal	Target Disease	Testing Conditions	References
DNA tetrahedron	Hydrogen ions and superoxide anion (O_2_^•−^)	7.2 nM for O_2_^•−^	fluorescence	inflammation, neurodegenerative diseases, and cancer	living cells	[122]
	TK1 mRNA	3.2 nM; 0.33 nM	fluorescence	cancer	living cells	[130,131]
	TK1 mRNA, GalNac-T mRNA, C-myc mRNA	3.1 nM for C-myc mRNA; 1.2 nM for TK1 mRNA; 3.2 nM for GalNAc-T mRNA	fluorescence	cancer	living cells	[132]
	TK1 mRNA	3.3 pM	fluorescence	cancer	living cells	[133]
	miRNA-21	0.03 fmol/10 μg_RNA_ for CD; 0.12 fmol/10 μg_RNA_ for luminescence	plasmonic circular dichroism (CD); luminescence	cancer	living cells	[134]
	BRCA1 DNA	10 fM	colorimetric	breast cancer	fetal calf serum	[136]
	HIV-related DNA	48 fM	surface plasmon resonance	acquired immunodeficiency syndrome (AIDS)	C57 wild type mice tail total DNA	[135]
	miRNA21; miRNA155; miRNA196a; miRNA210	10 fM	electrochemical	cancer	human serum samples	[137]
	miRNA (hsa-let-7a)	50 aM	electrochemical	asthma	cell lysates and fetal bovine serum	[138]
	DNA methyltransferase	0.045 U mL^–1^	fluorescence	cancer	human serum	[139]
	DNA methyltransferase	0.03 U mL^–1^	electrochemical	cancer	human serum	[143]
	Pneumococcal surface protein A (PspA) peptide	0.218 ng mL^−1^	electrochemical	pneumonia	human samples from nasal cavity, mouth and axilla	[145]
	Tumoral hepatocellular exosomes	2.09 × 10^4^ mL	electrochemical	cancer	isolated HepG2 hepatocelular exosomes	[145]
	Tumor cells	4 MCF-7 cancer cells	electrochemical	cancer	cell culture medium	[146]
DNA prism	ATP	0.03 mM	fluorescence (FRET)	hypoxia, ischemia, Parkison’s disease, some malignant cancers	living cells	[124]
DNA tweezer	miRNA-21	0.03 fM	electrochemiluminescence	cancer	living cells	[147]
DNA hydrogels	Tumor cells	<10 cancer cells	fluorescence	cancer	living cells	[148]
DNA origami	Zika-specific artificial DNA and RNA	-	fluorescence	zika infection	human blood serum	[149]
	*Plasmodium falciparum* lactate dehydrogenase (PfLDH)	500 nM	AFM	malaria	blood plasma	[150]
	Hepatitis B genotyping	10 pM	AFM	viral hepatitis	clinical hepatitis B virus DNA samples	[151,152]

**Table 2:** DNA-based nanostructures designed for deliver therapeutic cargo inside cells. *

DNA Nanostructure	Cargo	Functionalization/Chemical Modifications	Responsive/Specific	Therapy	Target	Testing Conditions	References
DNA icosahedron	DOX	MUC1 aptamer	Cells with MUC1 receptors	chemotherapy	cancer	in vitro	[160]
	Dehydroepiandrosterone (DHEA)	photoactivatable dextran–DHEA conjugate	photoresponsive	chemotherapy	activate neurons	in vivo	[114]
DNA tetrahedron	DOX	d-sugar DNA TDN and l-sugar DNA TDN	--	chemotherapy	cancer	in vivo	[165]
	Floxuridine oligomers	floxuridine oligomers- and cholesterol-conjugated ODNs	--	chemotherapy	colorectal cancer	in vitro	[171]
	siRNAs	tumour-targeting ligands and 2‘-O-methyl-ODNs	--	gene therapy	cancer	in vivo	[187]
	CpG ODNs and streptavidin	biotin-CpG ODNs, CpG ODNs and phophorothioate ODNs	--	immunotherapy	--	in vivo	[188]
DNA polyhedra	Floxuridine	floxuridine-conjugated ODNs	--	chemotherapy	cancer	in vivo	[170]
DNA nanotubes	DOX	--	--	chemotherapy	breast cancer	in vitro	[161,162]
	DOX	biotin/streptavidin-conjugated Qdot 655	--	chemotherapy	cancer	in vivo	[164]
	CpG ODNs	CpG-conjugated ODNs	--	immunotherapy	--	in vivo	[189]
Rod-like DNA origami	Daunorubicin	--	--	chemotherapy	leukemia model	in vitro	[163]
Triangle DNA origami	DOX	--	--	chemotherapy	breast cancer	in vitro	[162]
	DOX	biotin/streptavidin-conjugated Qdot 655	--	chemotherapy	cancer	in vivo	[164]
	DOX and p53 gene	MUC1 aptamers	cells with MUC1 receptors and redox sensitive	chemotherapy and gene therapy	breast cancer	in vivo	[203]
	DOX and shRNA	MUC1 aptamers	cells with MUC1 receptors and redox sensitive	chemotherapy and gene therapy	cancer	in vivo	[204]
Square DNA origami	DOX	biotin/streptavidin-conjugated Qdot 655	--	chemotherapy	cancer	in vivo	[164]
	Floxuridine oligomers	floxuridine oligomers- and cholesterol-conjugated ODNs	--	chemotherapy	colorectal cancer	in vitro	[171]
	Thrombin	AS1411 aptamers	responsive to nucleolin	protein therapy	ovarian cancer and melanoma	in vivo	[214]
Hexagonal DNA barrel	Antibody to human CD33 and antibody to human CDw328 Fab′ fragments	41t-, TE17-, and sgc8c aptamers	responsive to biological cues	protein therapy	cancer	in vitro	[81]
Spherical nucleic acids (SNAs)	BKM120	DNA-hexaethylene conjugates	--	chemotherapy	chronic lymphotic leukemia	in vivo	[166]
	siRNAs	AuNPs functionalized wit siRNAs	--	gene therapy	glioblastoma multiforme	in vivo	[193]
	CpG ODNs	AuNPs functionalized wit siRNAs	--	immunotherapy	lymphoma/liver fibrosis	in vivo	[195]
DNA prism	Antisense ODNs	phosphorothioated antisense ODNs	--	gene therapy	cancer	in vitro	[190]
	siRNAs	LNA- and phophorothioated-ODNs, hexaethylene glycol insertions, antisense, and siRNA ODNs	--	gene therapy	cancer	in vitro	[191]
Triangular rung units siRNA	siRNAs	sgc8c- and sgc4f aptamers	cell-specific	gene therapy	cancer	in vivo	[192]
DNA/RNA nanoflowers or nanoclews	DOX	PEG-folic acid-conjugated and embedded Dnase I nanocapsules	pH- responsive	chemotherapy	cancer	in vitro	[179]
	siRNAs	electrostactically coated with polyethylenimine (PEI)	--	gene therapy	cancer	in vivo	[196]
	siRNAs	electrostactically coated with thiolated glycol chitosan	redox sensitive	gene therapy	cancer	in vivo	[199]
	Multi-siRNAs	electrostactically coated with polyethylenimine (PEI)	--	gene therapy	cancer	in vitro	[200]
	CpG ODNs	anti-PD-1 antibody	bioresponsive to wound sites	immunotherapy	cancer	in vivo	[201]
	CpG ODNs, shRNA and peptide therapeutics	electrostactically coated with PEG-grafted polypeptide copolymers	tumor-specific antitumor immunity	immunotherapy	colorectal cancer	in vivo	[202]
	Cas9 protein and sgRNA	electrostactically coated with polyethylenimine (PEI)	--	gene therapy	cancer	in vivo	[215]
	Cytokines	cytokine TRAIL was loaded into the Ni^2+^ modified DNA nanoclew cores via Ni^2+^ -polyhistidine affinity	degradable by phospholipase A2	protein therapy	colorectal cancer	in vitro	[216]

* In this table is summarized the main features of the designed DNA nanodevices not including the fluorescent dyes that are commonly attached to perform studies such as confocal microscopy or flow cytometry among others.
